# Cardiac diagnostic work-up for atrial fibrillation after transient ischaemic attacks in England and Wales: results from a cross-sectional survey

**DOI:** 10.1136/bmjopen-2016-012714

**Published:** 2016-11-10

**Authors:** Olivia Geraghty, Eleni Korompoki, Filippos T Filippidis, Anthony Rudd, Roland Veltkamp

**Affiliations:** 1 Department of Stroke Medicine, Imperial College London, London, UK; 2 Department of Primary Care and Public Health, Imperial College London, London, UK; 3 Department of Stroke Medicine, Guy's and St Thomas’ NHS Foundation Trust, St Thomas’ Hospital, London, UK; 4 National Clinical Director of Stroke, NHS England; 5 London Stroke Clinical Director, London, UK

**Keywords:** Atrial fibrillation; Transient ischaemic attack; Ischaemic stroke; ECG, STROKE MEDICINE

## Abstract

**Objectives:**

Transient ischaemic attacks (TIAs) are an important precursor of stroke. Atrial fibrillation (AF) is among the most dangerous aetiologies shared between TIAs and strokes. Detection of AF after TIAs is essential for the initiation of oral anticoagulants. We aimed to identify variations in the use of cardiac investigations used to detect AF and cardiac pathology in patients with TIA in the UK.

**Setting:**

All TIA clinical leads in England and Wales received an invitation by email to participate in an online survey in February 2015. The questionnaire consisted of 36 multiple choice questions covering the domains: (1) general information about stroke units, (2) ECG diagnostics and cardiologic work-up and (3) management of AF.

**Results:**

146 survey invitations were sent. The response rate was 40% (n=59). Diagnosis of AF largely depends on medical history and 12-channel ECG which is performed in the vast majority of patients with TIA (>75%) in 94.1% of the TIA services. Many patients with TIA either do not receive 24-hour Holter recording (requested regularly in 42% of the services) or only after considerable delay (>2 weeks). Prolonged event recording is only rarely performed (16%). Only about half of patients with TIA undergo echocardiography. Cranial imaging in patients with TIA is mainly performed as CT (62%). The majority of TIA clinics rapidly initiate anticoagulation in TIA patients with AF (81.6%) preferably using new oral anticoagulants (75.5%).

**Conclusions:**

Significant variation in the cardiac diagnostic work-up following TIA exists regarding the use of particular detection techniques and the duration of cardiac ECG monitoring. Only limited resources are allocated to cardiac evaluation. In addition to research establishing the optimal ECG technique for patients with TIA, healthcare delivery programmes are needed to ensure proper management to prevent strokes.

**Strengths and limitations of this study**

- The first survey specifically in patients with transient ischaemic attack (TIA) rather than in mixed cohort of patients with ischaemic stroke and TIA.
- Participants are TIA clinical leads with insight into their service.
- Survey of opinions of practice rather than an audit of actual data.
- Moderate response rate to the survey.
- Reflection of practices within the National Health Service in England and Wales, which cannot be generalised to all international healthcare practices.

## Introduction

Transient ischaemic attacks (TIAs) are a common neurologic emergency with an incidence rate of 0.05–7.20 per 1000 person years in Europe that increases markedly with age.^1^ In terms of their origin and nature, TIAs are nowadays considered as part of the spectrum of clinical manifestations of ischaemic stroke (IS) rather than as a separate entity. The reason for this is the similar rate of recurrence as well as common aetiological pathways leading to stroke and TIA. Atrial fibrillation (AF) is a common and treatable risk factor for stroke.^2^ Although a fifth of patients with stroke have known AF, probably at least as many have unrecognised paroxysmal AF.^3^ ^4^ Similarly, cardioembolism accounts for 20–26% of all TIAs with AF representing the leading cause of embolism.^5^ ^6^ Detection of AF after TIAs is crucial since oral anticoagulants (OACs) can reduce the risk of stroke in TIA patients with AF by two-thirds.^7^ ^8^

The rate of detection of paroxysmal AF depends on the technique and duration of ECG monitoring as well as on the characteristics of the investigated patient cohort (eg, age).^9^ The yield of AF detection using inpatient cardiac monitoring is between 3.8% and 6.5% in patients with stroke and TIA without a previous diagnosis of AF and without AF on admission ECG.^10^ ^11^ Extensive outpatient monitoring using non-invasive or implanted event recorders newly detects AF in up to 16.9% of patients with stroke. However, data specifically addressing patients with TIA are lacking.^4^

By which methods and in which sequence patients with TIA should be investigated is the matter of an ongoing debate.^12^ Recent American guidelines for secondary stroke prevention recommend 30-day ECG event recording in patients with stroke or TIA of unexplained cause based on findings of the EMBRACE trial.^13^ ^14^ In contrast, European and UK guidelines are less definitive regarding the duration and choice of ECG monitoring techniques for AF detection after TIA.^15^ ^16^ Moreover, the settings for diagnostic work-up of strokes and TIAs differ substantially. In many healthcare systems including the UK, TIAs are evaluated on an outpatient basis whereas strokes are admitted to stroke units with bedside monitors.^17^ Even if patients with TIA are hospitalised, their length of stay on monitored stroke units is shorter.^18^ While there is growing evidence guiding diagnostic work-up for detection of AF in patients with stroke, the yield of cardiac work-up in patients with TIA has not been studied specifically. This uncertainty is likely to affect cardiac evaluation in routine practice. National Clinical Guidelines for TIA management in England require all high-risk patients (ABCD2 score of 4 and above) to be assessed and management started within 24 hours of onset of symptoms and all other patients within 1 week. The median number of neurovascular clinics provided by each hospital per month is 20 and most are set up to provide specialist assessment, and brain and carotid imaging as an outpatient on the day the patient is seen.^19^ ^20^ However, routine practice for cardiac evaluation of patients with TIA in the UK is largely unknown.

Hence, we performed an online survey among all TIA service leads in England to determine the current diagnostic and therapeutic practice in the UK.

## Methods

We conducted an anonymous cross-sectional survey during February and March 2015, using a standardised questionnaire via survey monkey (online data supplement) in order to assess the current cardiac diagnostic work-up after TIA in the UK. The questionnaire was sent to all National Health Service (NHS) TIA clinical leads in England and Wales.

10.1136/bmjopen-2016-012714.supp1 Supplementary data

All trust TIA leads were identified by the following steps: (1) web-searching (http://www.nhs.uk/servicedirectories) and identifying list of all trusts in England and Wales, (2) trusts not providing stroke services (eg, children's hospitals, women's hospitals) were excluded from the list and (3) lead physicians at TIA clinics, stroke units and geriatric departments were contacted by phone. Phone numbers were identified either online or by contacting the switchboard operators at the trusts. For each trust, the email of the lead consultant for the TIA service was requested. The clinical leads were asked for their own impression of the diagnostic evaluation of AF following TIA at their service. The survey was based on a questionnaire sent online to all TIA clinical leads.

The research questionnaire and study protocol were approved by Research and Development (R&D) at Imperial College London. No further ethical approval was required for this type of research (anonymous online survey). Responses were anonymised. In addition, no individual patient data were collected. The survey questionnaire aimed to assess each UK trust's practice in the diagnostic work-up of AF following TIA. The questionnaire comprised 36 questions and covered the following main categories: (1) general information about stroke units, (2) AF diagnostics and work-up and (3) management of AF. The questions were based on a previously used online tool assessing a similar research question in acute stroke units in Germany.^18^ All answers were multiple choice, multiple selection was not allowed. The questionnaire was pilot tested with four consultants within our stroke service. Responders were invited to estimate their perception about cardiac work-up in TIA clinics as a predefined percentage. In total, three reminders were sent to all TIA clinical leads. Descriptive results are presented as percentage.

## Results

### Characteristics of participating trusts

A total of 146 invitations to participate in our survey were sent. The response rate after sending three reminders was 40% (n=59). Of the clinicians who participated, 44 (74.5%) were stroke physicians, 5 (8.5%) were neurologists and 10 (17%) belonged to other specialities. A hyperacute stroke unit exists in 75% of responding trusts providing a TIA service. A 7-day TIA service is provided in 32 (54.5%) of responding trusts. The estimated number of suspected patients with TIA seen per year is >500 in 50% of trusts. The majority of trusts (75%) admit <10% of patients with TIA to inpatient services.

### Use of different modalities of ECG monitoring

**Figure 1:**
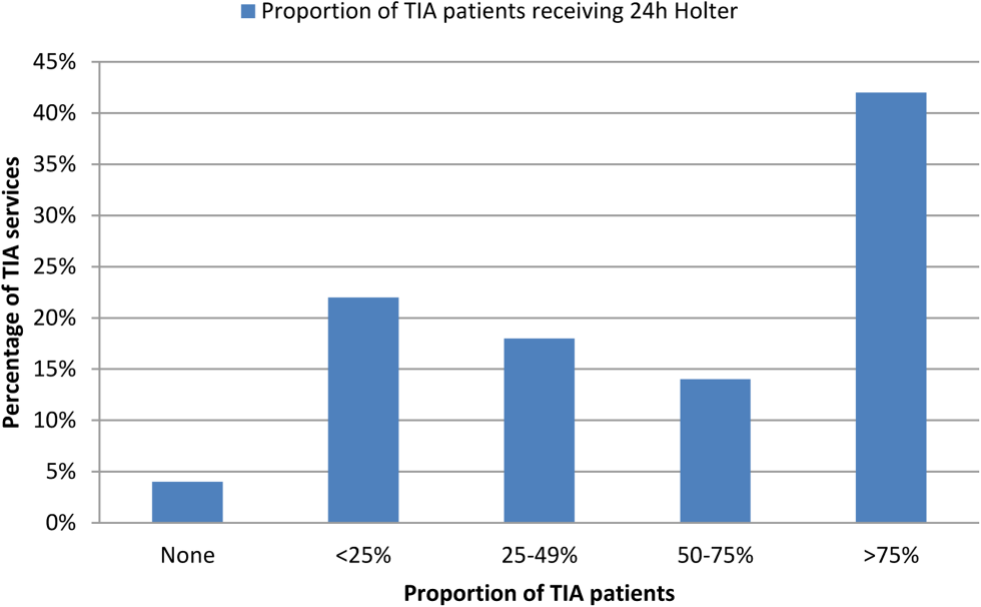
Estimated proportion of patients with TIA without known AF that receive 24-hour ECG monitoring in TIA services. AF, atrial fibrillation; TIA, transient ischaemic attack.

**Figure 2:**
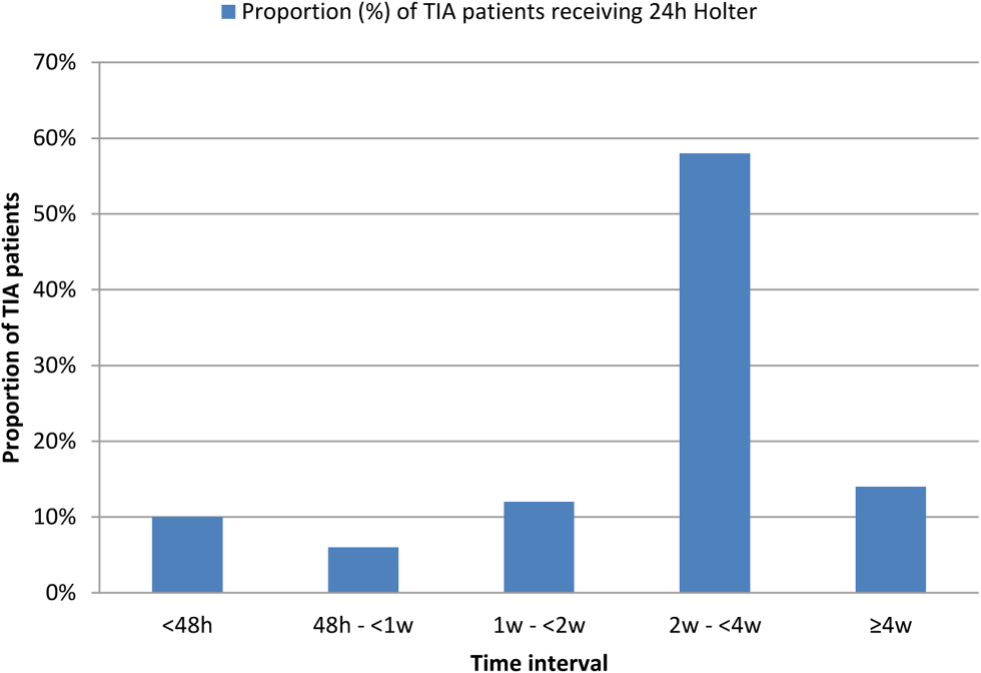
Estimated proportion of patients receiving 24-hour Holter monitoring within a given time interval after initial clinical assessment in TIA services. TIA, transient ischaemic attack.

A 12-lead ECG is performed in >75% of patients in the vast majority (94.1%) of trusts (table 1). In contrast, 24-hour ECG monitoring in patients without previously known AF is requested in >75% of patients by only less than half (42%) of services (figure 1). The average time interval between clinical assessment in the TIA clinic and 24-hour ECG monitoring was >2 weeks in the majority (72%) of trusts (figure 2).

**Table 1:** Proportion of patients with transient ischaemic attack receiving different ECG modalities and echocardiographic techniques

	ECG modalities				ECHO techniques	
	12-lead ECG	24-hour Holter	External event recorder	Invasive monitoring	TTE	TOE
None	0% 0	4% 2	38% 18	43% 21	2% 1	18% 9
<25%	4% 2	22% 11	48% 23	53% 26	40% 21	80% 39
25–49%	4% 2	18% 9	8% 4	4% 2	26% 13	0% 0
50–75%	2% 1	14% 7	0% 0	0% 0	16% 8	0% 0
>75%	94% 48	42% 21	6% 3	0% 0	14% 7	2% 1

ECHO, echocardiographic; TOE, transoesophageal echocardiogram; TTE, transthoracic echocardiography.

**Figure 3:**
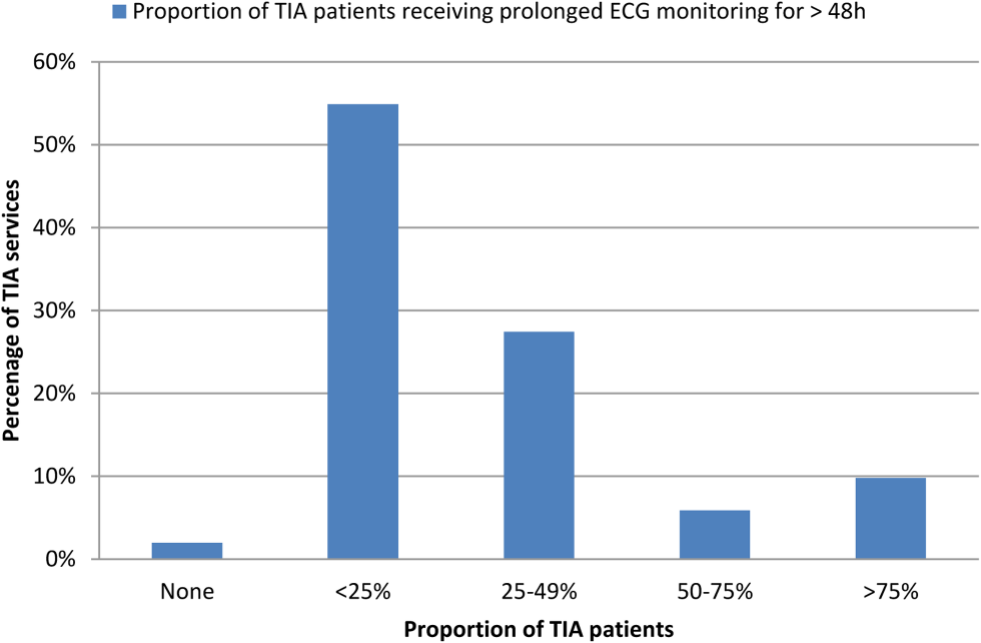
Estimated proportion of patients with TIA without known AF that receive prolonged ECG monitoring for ≥ 48 hours in TIA services. AF, atrial fibrillation; TIA, transient ischaemic attack.

Prolonged ECG monitoring exceeding 48 hours is infrequently performed (>50% of patients in only 16% of trusts) (figure 3). Only 7.8% of TIA leads considered prolonged monitoring ≥7 days for investigation of TIA, whereas 45% of respondents considered <72 hours as an appropriate duration for ECG monitoring for detection of AF following TIA.

A total of 37.5% of respondents would never consider an external event recorder in patients with TIA, and 43% of respondents would never consider an implantable loop recorder device for detection of AF in their patients with TIA (table 1).

Reasons that prompt physicians to perform more prolonged ECG monitoring in the event of normal 24-hour Holter ECG are presented in table 2. Neuroimaging characteristics, that is, multiple territory embolic-looking infarcts were the most prevalent reason for more prolonged monitoring followed by cryptogenic stroke and younger age (table 2).

**Table 2:** Reasons in rank order (1: least important, 7: most important) that prompt physicians to do more prolonged ECG monitoring for occult paroxysmal AF detection in the event of a normal 24-hour ECG

	1 (least important)	2	3	4	5	6	7 (most important)	Total	Score
Age <65 years	21.28% 10	10.64% 5	19.15% 9	21.28% 10	8.51% 4	4.26% 2	14.89% 7	47	4.43
Large cortical infarction on imaging	4.44% 2	24.44% 11	6.67% 3	20.00% 9	15.56% 7	20.00% 9	8.89% 4	45	3.87
Multiple territory embolic infarcts	41.30% 19	8.70% 4	10.87% 5	4.35% 2	0.00% 0	4.35% 2	30.43% 14	46	4.52
Cryptogenic stroke	12.77% 6	19.15% 9	23.40% 11	12.77% 6	14.89% 7	17.02% 8	0.00% 0	47	4.51
High NIHSS score	15.56% 7	13.33% 6	4.44% 2	6.67% 3	8.89% 4	17.78% 8	33.33% 15	45	3.33
Left atrium size	2.13% 1	8.51% 4	17.02% 8	25.53% 12	27.66% 13	17.02% 8	2.13% 1	47	3.72
History of palpitations	4.26% 2	17.02% 8	19.15% 9	12.77% 6	23.40% 11	14.89% 7	8.51% 4	47	3.87

NIHSS, National Institutes of Health Stroke Scale.

### Cardiac and brain imaging

Transthoracic echocardiography is performed in >50% of patients by only 30% of respondents (table 1). The average time interval between clinical evaluation and the echocardiogram exceeds 2 weeks in 70% of trusts. Transoesophageal echocardiography is rarely considered in the diagnostic work-up of patients with TIA (≤25% of patients in 97% of services) (table 1).

Cranial CT is used as the first-line imaging in >75% of patients in 62% of trusts. In contrast, MRI is used as the first-line brain imaging in >50% of patients by only 19.5% of responding trusts. Among patients with TIA without infarcts on CT, MRI is only performed in a fraction of patients (>50% of patients receive an additional brain MRI in only 17% of trusts).

### Aspects of therapeutic management

More than 75% of patients with newly diagnosed AF are recommended to start an OAC at TIA clinics in 95.7% of trusts. For those patients who are considered for OAC, this is started in the TIA clinic in 81.6% of cases. More than 75% of patients are offered a follow-up appointment by 58.3% of trusts. Novel oral anticoagulants (NOAC) are considered the first choice in stroke prevention among the majority (75.5%) of respondents.

Scores assessing risk of thromboembolism in AF and risk of major bleedings (ie, CHA_2_DSVA_2_Sc and HAS BLED score, respectively) are used by the majority of services (63%), whereas 20% do not use any risk stratification scores. Only a small proportion of patients with newly detected AF are referred to a cardiologist (<25% of patients in 73.4% of trusts). According to the respondents, the factors most likely to trigger specialist referral are structural abnormalities on echocardiography.

## Discussion

The major findings of our survey are that (1) detection of AF largely depends on history and 12-lead ECG in patients with TIA, (2) many patients with TIA either do not receive 24-hour Holter recording or do so only after considerable delay, (3) extensive event recording in patients with TIA is very unusual, (4) echocardiography is performed only in about half of patients with TIA and usually with considerable delay, (5) cranial imaging in patients with TIA is mainly performed as CT and (6) TIA clinics rapidly initiate anticoagulation in TIA patients with AF with a preference for NOACs.

Our study highlights a substantial variation in the cardiac diagnostic work-up of patients with TIA in England and Wales. The vast majority of this variation is around the methods used for detection and around the duration of ECG monitoring. Despite existing evidence of the usefulness of extensive ECG monitoring for AF detection from observational and randomised trials,^4^ ^9–11^ ^13^ ^21^ 12-lead ECG is the only consistently used technique by TIA clinics in England and Wales. Even 24-hour Holter ECG, which is considered the routine diagnostic standard for AF detection after stroke^16^ ^22^ despite its limited sensitivity, is only requested by about half of services. Considering further limitations in the availability of this basic diagnostic test, this means that opportunities for stroke prevention are missed. However, we have to acknowledge that prolonged monitoring may not be required in all patients with TIA but only in selected patients with specific clinical and neuroimaging characteristics. Interestingly in our study, multiple embolic infarcts in neuroimaging, cryptogenic stroke and younger age were the most prevalent reasons for prolonged ECG monitoring beyond 24 hours.

Echocardiography is not routinely performed in many TIA clinics which may reflect that no recommendations relating to the use of echocardiography in the assessment of the first episode of stroke and TIA were made in the National Institute for Health and Care Excellence (NICE) acute stroke and TIA guideline or the Department of Health National Stroke Strategy.^23^ The sensitivity for detection of left ventricular thrombus is similar in transthoracic compared with transoesophageal echocardiography but detection of patent foramen ovale, cardiac vegetations and left atrial thrombus by transoesophageal echocardiography is superior.^24^

The current practice of neuroimaging in patients with TIA in England and Wales does not match current NICE guidelines.^22^ According to NICE, MRI should be performed as the first-line imaging in patients with TIA. About a third of patients with a recent TIA have evidence of infarction on diffusion-weighted MR imaging (DWI). DWI lesions are seen more often in TIA patients with AF and are associated with a higher incidence of stroke recurrence at 90 days when compared with DWI-negative TIAs.^25^ ^26^ In these patients, imaging-based assessment of stroke mechanisms including an embolic stroke pattern is frequently possible and this aetiological classification may affect the extent of cardiac investigations. Interestingly, the implications of imaging findings on secondary prevention are currently investigated in randomised trials of embolic strokes of undetermined source (NCT02313909, NCT02239120, NCT02427126). However, the findings of these trials will not immediately affect secondary stroke prevention in the majority of patients with TIA without infarcts on imaging.

In addition to the challenges in diagnosing AF, therapeutic obstacles to OAC prescribing exist in the community. These include lack of ownership of the problem by primary and secondary care, safety concerns and differences between physicians’ and patients’ expectations regarding the management of AF.^22^ A reassuring result of our survey is that the majority of TIA services in England and Wales initiate anticoagulant prescribing and also arrange follow-up. Interestingly, TIA services in England and Wales frequently choose NOACs in this setting which is in contrast to the lower overall prescription of these drugs in the UK compared with other European countries.^27^ Presumably, this preference for NOACs in TIA clinics may reflect the rapid onset of action of NOACs and the low risk of intracranial haemorrhage in the absence of large brain infarcts. On the other hand, this practice has important implications for the management of patients with TIA particularly in outpatient clinics as mechanisms for adequate counselling regarding oral anticoagulation in AF have to be ascertained in accordance with NICE guidance.^28^ This includes the use of scores to assess the risk of thromboembolism and bleeding, respectively.^28^ Our survey suggests that only part of the services use these tools in daily practice so far. Moreover, pathways have to be established for adequate follow-up.^28^

Our study has several limitations. First, it is a survey of opinions of practice rather than an audit of actual data. However, those surveyed are TIA clinical leads with insight into their service, and the variation in practice suggests that the appropriate diagnostic strategy is generally unclear at present. Second, the response rate of 40% to our survey was only moderate and may have led to bias. Owing to our methods (use of anonymous online questionnaire sent via survey monkey), a comparison between responding and non-responding centres that could indicate whether our sample was representative cannot be made. However, the characteristics of the services provided by the responding sites reflect what is known about typical neurovascular services in England. The majority are delivered by stroke physicians with a smaller proportion by neurologists. Most run clinics at least 5 days a week and most are set up to establish the likely diagnosis and initiate appropriate secondary prevention on the day the patient is seen. Third, our study reflects practices within the NHS in England and Wales, which cannot be generalised to all international healthcare practices some of which evaluate TIAs as inpatients. However, the currently limited evidence for the best cardiac monitoring strategy for AF detection in patients with TIA is likely to affect the extent of cardiac testing in most parts of the world. Fourth, TIA leads were not asked whether particular clinical symptoms on presentation including presumed cortical syndromes serve as a guidance for decision-making regarding further investigations. This is another limitation considering that specific clinical features (eg, cortical symptoms) may indicate a cardioembolic mechanism. Finally, our findings are descriptive in nature and potential causes for limited use of certain diagnostic tests such as lack of resources rather than medical judgement were not explored.

In conclusion, our study suggests that only limited resources are allocated to cardiac evaluation in patients with TIA, and that the practice varies substantially among services in the UK. Research studies establishing the optimal ECG monitoring technique specifically for patients with TIA as well as healthcare delivery programmes are needed to ensure effective stroke prevention in patients with TIA.

## References

[1] Kokubo Y Epidemiology of transient ischemic attack. Front Neurol Neurosci 2014;33:69–81. 10.1159/000351892 24157557

[2] Wolf PA, Abbott RD, Kannel WB Atrial fibrillation as an independent risk factor for stroke: the Framingham study. Stroke 1991;22:983–8. 10.1161/01.STR.22.8.983 1866765

[3] Andrew NE, Thrift AG, Cadilhac DA The prevalence, impact and economic implications of atrial fibrillation in stroke: what progress has been made? Neuroepidemiology 2013;40:227–39. 10.1159/000343667 23364221

[4] Sposato LA, Cipriano LE, Saposnik G Diagnosis of atrial fibrillation after stroke and transient ischaemic attack: a systematic review and meta-analysis. Lancet Neurol 2015;14:377–87. 10.1016/S1474-4422(15)70027-X 25748102

[5] Amort M, Fluri F, Weisskopf F Etiological classifications of transient ischemic attacks: subtype classification by TOAST, CCS and ASCO--a pilot study. Cerebrovasc Dis 2012;33(6): 508–16. 10.1159/000337236 22538846

[6] Hayashi T, Seahara Y, Kato Y Clinical characteristics of cardioembolic transient ischemic attack: comparison with noncardioembolic transient ischemic attack. *J Stroke Cerebrovasc Dis* 2014;23:2169–73. 10.1016/j.jstrokecerebrovasdis.2014.04.005 25088173

[7] Secondary prevention in non-rheumatic atrial fibrillation after transient ischaemic attack or minor stroke. EAFT (European Atrial Fibrillation Trial) Study Group. Lancet 1993;342:1255–62. 7901582

[8] Ntaios G, Papavasileiou V, Diener HC Nonvitamin-K-antagonist oral anticoagulants in patients with atrial fibrillation and previous stroke or transient ischemic attack: a systematic review and meta-analysis of randomized controlled trials. Stroke 2012;43:3298–304. 10.1161/STROKEAHA.112.673558 23150654

[9] Kishore A, Vail A, Majid A Detection of atrial fibrillation after ischemic stroke or transient ischemic attack: a systematic review and meta-analysis. Stroke 2014;45:520–6. 10.1161/STROKEAHA.113.003433 24385275

[10] Kallmünzer B, Breuer L, Hering C A structured reading algorithm improves telemetric detection of atrial fibrillation after acute ischemic stroke. Stroke 2012;43:994–9. 10.1161/STROKEAHA.111.642199 22308240

[11] Rizos T, Guntner J, Jenetzky E Continuous stroke unit electrocardiographic monitoring versus 24-hour Holter electrocardiography for detection of paroxysmal atrial fibrillation after stroke. Stroke 2012;43:2689–94. 10.1161/STROKEAHA.112.654954 22871678

[12] Diener HC To monitor or to not monitor for paroxysmal atrial fibrillation after transient ischemic attack or stroke: this is the question. Stroke 2014;45:355–6. 10.1161/STROKEAHA.113.004036 24385280

[13] Gladstone DJ, Spring M, Dorian P Atrial fibrillation in patients with cryptogenic stroke. N Engl J Med 2014;370:2467–77. 10.1056/NEJMoa1311376 24963566

[14] Kernan WN, Ovbiagele B, Black HR Guidelines for the prevention of stroke in patients with stroke and transient ischemic attack: a guideline for healthcare professionals from the American Heart Association/American Stroke Association. Stroke 2014;45:2160–236. 10.1161/STR.0000000000000024 24788967

[15] Guidelines for management of ischaemic stroke and transient ischaemic attack 2008. Cerebrovasc Dis 2008;25:457–507. 10.1159/000131083 18477843

[16] Royal College of Physicians. National Clinical Guideline for Stroke. Secondary National Clinical Guideline for Stroke 2012 https://www.rcplondon.ac.uk/guidelines-policy/stroke-guidelines

[17] Jauch EC, Saver JL, Adams HP Jr Guidelines for the early management of patients with acute ischemic stroke: a guideline for healthcare professionals from the American Heart Association/American Stroke Association. Stroke 2013;44:870–947. 10.1161/STR.0b013e318284056a 23370205

[18] Rizos T, Quilitzsch A, Busse O Diagnostic work-up for detection of paroxysmal atrial fibrillation after acute ischemic stroke: cross-sectional survey on German stroke units. Stroke 2015;46:1693–5. 10.1161/STROKEAHA.115.009374 25931467

[19] Intercollegiate Stroke Working Party. National Clinical Guidelines for Stroke 2012 https://www.rcplondon.ac.uk/file/1299/download?token=mcyQFjEq

[20] Sentinel Stroke National Audit Programme. Acute Organisational Audit 2014 https://www.strokeaudit.org/results/Organisational.aspx

[21] Sanna T, Diener HC, Passman RS Cryptogenic stroke and underlying atrial fibrillation. N Engl J Med 2014;370:2478–86. 10.1056/NEJMoa1313600 24963567

[22] National Institute for Health and Clinical Excellence. Stroke: diagnosis and initial management of acute stroke and transient ischaemic attack (TIA). Secondary stroke: diagnosis and initial management of acute stroke and transient ischaemic attack (TIA) 2008 https://www.nice.org.uk/guidance/cg68

[23] Department of Health. National Stroke Strategy. Secondary National Stroke Strategy 2007 http://clahrc-gm.nihr.ac.uk/wp-content/uploads/DoH-National-Stroke-Strategy-2007.pdf

[24] Doufekias E, Segal AZ, Kizer JR Cardiogenic and aortogenic brain embolism. J Am Coll Cardiol 2008;51:1049–59. 10.1016/j.jacc.2007.11.053 18342221

[25] Redgrave JN, Coutts SB, Schulz UG Systematic review of associations between the presence of acute ischemic lesions on diffusion-weighted imaging and clinical predictors of early stroke risk after transient ischemic attack. Stroke 2007;38:1482–8. 10.1161/STROKEAHA.106.477380 17379821

[26] Calvet D, Touze E, Oppenheim C DWI lesions and TIA etiology improve the prediction of stroke after TIA. Stroke 2009;40:187–92. 10.1161/STROKEAHA.108.515817 18988917

[27] Le Heuzey JY, Ammentorp B, Darius H Differences among western European countries in anticoagulation management of atrial fibrillation. Data from the PREFER IN AF registry. Thromb Haemost 2014;111:833–41. 10.1160/TH13-12-1007 24651882

[28] National Institute for Health and Care Excellence. Atrial fibrillation: management. Secondary atrial fibrillation: management 2014 https://www.nice.org.uk/guidance/cg180

