# Supplementary material for: Cardiac diagnostic work-up for atrial fibrillation after transient ischaemic attacks in England and Wales: results from a cross-sectional survey
Source: BMJ Open. 2016 Nov 10;6(11):e012714. doi: 10.1136/bmjopen-2016-012714 (PMC5129110; doi:10.1136/bmjopen-2016-012714)
Supplement: Supplementary data [file bmjopen-2016-012714supp.pdf]

SUPPLEMENTARY DATA

Survey to assess diagnostic work up of all patients following TIA in the U.K

General Information regarding your stroke unit:

1. Please select your job description
   - Stroke Physician
   - Stroke Neurologist
   - General Medicine Physician
   - Other specify ________________________

2. Does your Hospital have a Hyperacute stroke unit?
   - Yes
   - No

3. Which type of hospital trust/authority is your clinic located?
   - University Hospital NHS Trust
   - NHS trust
   - Other: specify ________________________

4. Can you provide a reasonable estimate of the absolute number of TIA patients that are seen at your trust per year?
   - <100
   - 100-<250
   - 250-499
   - 500—749
   - 750 - 999
   - ≥1000

5. What is the estimated percentage of your TIA patients admitted to your inpatient service?
   - none
   - <10%
   - 10-<30%
   - 30->50%
   - ≥50%

6. How many days per week does your TIA clinic provide its services?
   - 5 days a week
   - 7 days a week
   - Other (please specify)

Information with regards to AF diagnosis and work up at your service

7. In your clinical practice, what estimated percentage of TIA patients receive a 12 lead ECG

- None
- <25%
- 25-49%
- 50-75%
- >75%

8. What is the estimated proportion of TIA patients without known AF that receive 24h-ECG monitoring?

- None

- <25%
- 25-49%
- 50-75%
- >75%

9. What is the average time interval between assessment of TIA and 24 hour ECG monitoring?

- <48 hours
- 48 hours - <1 week
- 1 week - <2 weeks
- 2 weeks - <4 weeks
- ≥4 weeks

10. What is the estimated proportion of TIA patients without known AF that receive prolonged ECG monitoring (i.e. 48 hours or more)?

- None
- <25%
- 25-49%
- 50-75%
- >75%

11. What is the average length of prolonged ECG monitoring considered for investigation of TIA?

- <72 hours
- 72 hours -7 days
- >7 days

12. What is the estimated proportion of TIA patients without known AF that receive a Transthoracic echocardiogram (ECHO)?

- None
- <25%
- 25-49%
- 50-75%
- >75%

13.What is the average time interval between assessment of TIA and transthoracic ECHO

- <48 hours
- 48 hours - < 1 week
- 1 week - <2 weeks
- 2 weeks - <4 weeks
- ≥4 weeks

14. What is the estimated proportion of TIA patients without known AF that receive a transoesophageal echocardiogram (TOE)?

- None
- <25%
- 25-49%
- 50-75%
- >75

15. What is the average time interval between assessment of TIA and transoesophageal ECHO?

- <48 hours
- 48 hours - 1 week
- 1 week - <2 weeks
- 2 weeks - <4 weeks
- ≥4 weeks

16. In your clinical practice, in what estimated proportion of patients would you consider an external event recorder device for the detection of occult atrial fibrillation in TIA patients?

- None
- <25%
- 25-49%
- 50-75%

- >75%

17. In your clinical practice, in what estimated proportion of patients would you consider an implantable event recorder device for the detection of occult atrial fibrillation in TIA patients?

- None
- <25%
- 25-49%
- 50-75%
- >75%

18. What is the average monitoring duration of your external monitoring devices?

- ≤7 days weeks
- 7 days - ≤ 4 weeks
- 4 weeks - ≤1 year
- > 1 year

19. What is the average time interval between patient admission and external recording devices?

- Within 1 week after the event
- 1- <4 weeks after the event
- 4- <12 weeks after the event
- ≥12 weeks after the event

20. What is the average monitoring duration of your implanted monitoring devices?

- ≤7 days weeks
- 7 days - ≤ 4 weeks
- 4 weeks - ≤1 year>1 year

21. What is the average time interval between patient admission and implanted recording devices?

- Within 1 week after the event
- 1 - <4 weeks after the event
- 4 -<12 weeks after the event
- ≥ 12 weeks after the event

22. What percentage of your TIA patients receives CT brain imaging at TIA clinic as first line imaging?

- None
- <25%
- 25-49%
- 50-75%
- >75

23. What percentage of your TIA patients receives MRI brain imaging at TIA clinic as first line imaging?

- None
- <25%
- 25-49%
- 50-75%
- >75

24. What percentage of “CT negative” TIA patients receives MRI brain imaging?

- None
- <25%
- 25-49%
- 50-75%

  >75%

25. In those patients with TIA of presumed other aetiology such as large artery disease, would you continue to investigate for occult atrial fibrillation with 24 hour ECG monitoring?

- Yes

- No

26. In your clinical practice, in the event of a normal 24 hour ECG recording, what clinical factors,(in rank order of importance), would prompt you to do more prolonged ECG monitoring to search for occult paroxysmal atrial fibrillation? Rank score 1(least important) to 7(most important factor)

- Age <65 years
- Large cortical infarction on imaging
- Multiple territory embolic looking infarcts on brain imaging
- Cryptogenic stroke
- High NIHSS score
- Left Atrium size
- History of palpitations

Management of atrial fibrillation:

27. What estimated percentage of TIA patients that are admitted as inpatients are admitted to a unit providing ECG telemetry?

- None
- <25%
- 25-49%
- 50-75%
- >75%

28. If a TIA patient is considered for oral anticoagulation, who starts this?

- TIA clinic
- General practitioner
- Anticoagulation clinic
- Other specify____________________
- Unsure

29. What is the estimated proportion of TIA patients who receive a follow-up clinic appointment?

- None
- <25%
- 25-49%
- 50-75%
- >75

30. What proportion of TIA patients who are anticoagulated are followed up in your clinic?

- None
- <25%
- 25-49%
- 50-75%
- >75

31. Which proportion of TIA patients with newly detected atrial fibrillation do you refer to a cardiologist?

- None
- <25%
- 25-49%
- 50-75%
- >75

32. In those patients with TIA of TOAST aetiology, i.e. large artery aetiology, would this affect the extent of the diagnostic work-up for paroxysmal AF?

- Yes
- No
- Unsure

33. What clinical factors would determine your referral to a cardiologist in a TIA patient with newly detected atrial fibrillation - tick more than one if necessary

- Rate control
- Difficult decision with anticoagulation
- Heart failure
- Structural abnormality on ECHO

34. Do you perform risk stratification scores on all patients who are considered for anticoagulation performed?

- Yes, both $CHADS_2VASC$ and HAS BLED scores
- Yes, only $CHADS_2VASC$
- Yes, but only HAS BLED
- No

35. What percentage of TIA patients that are newly diagnosed with AF are started or are recommended to start an anticoagulant therapy?

- None
- <25%
- 25-49%
- 50-74%
- >75%

36. Are new oral anticoagulants your first choice when considering anticoagulation in TIA patients with atrial fibrillation.

- Yes
- No
